# Pathogenic Mechanisms of Hypertrophic Cardiomyopathy beyond Sarcomere Dysfunction

**DOI:** 10.3390/ijms22168933

**Published:** 2021-08-19

**Authors:** Chun Chou, Michael T. Chin

**Affiliations:** 1 Department of Medicine, Tufts University School of Medicine, Boston, MA 02111, USA; chun.chou@tufts.edu; 2 Molecular Cardiology Research Institute, Tufts Medical Center, Boston, MA 02111, USA

**Keywords:** hypertrophic cardiomyopathy, pathological cardiac hypertrophy, sarcomere, cardiac myocyte, cardiac fibroblast, cardiac fibrosis, myocyte–fibroblast interaction, extracellular matrix

## Abstract

Hypertrophic cardiomyopathy (HCM) is the most common inherited cardiovascular disorder, affecting 1 in 500 people in the general population. Although characterized by asymmetric left ventricular hypertrophy, cardiomyocyte disarray, and cardiac fibrosis, HCM is in fact a highly complex disease with heterogenous clinical presentation, onset, and complications. While HCM is generally accepted as a disease of the sarcomere, variable penetrance in families with identical genetic mutations challenges the monogenic origin of HCM and instead implies a multifactorial cause. Furthermore, large-scale genome sequencing studies revealed that many genes previously reported as causative of HCM in fact have little or no evidence of disease association. These findings thus call for a re-evaluation of the sarcomere-centered view of HCM pathogenesis. Here, we summarize our current understanding of sarcomere-independent mechanisms of cardiomyocyte hypertrophy, highlight the role of extracellular signals in cardiac fibrosis, and propose an alternative but integrated model of HCM pathogenesis.

## 1. Introduction

Hypertrophic cardiomyopathy (HCM) is an autosomal dominant inherited disorder characterized by ventricular hypertrophy, frequently complicated by left ventricular outflow obstruction, diastolic heart failure, and sudden death [1,2]. Histologically, in addition to cardiomyocyte hypertrophy, cardiomyocyte disarray and cardiac fibrosis are often found. To date, multiple genome-wide association studies identified numerous genetic mutations associated with HCM phenotypes in large and unrelated cardiomyopathy families [3,4,5,6,7], involving genes encoding MYH7, MYL2, MYL3, MYBPC3, TNNT2, TNNI3, and TPM1. Among patients with sarcomere gene mutations, the most affected gene is *MYBPC3*, followed closely by *MYH7*, which together account for approximately 70% of patients in whom these mutations are found.

Despite extensive studies on the genetics of HCM, only 20–30% of patients with established clinical diagnosis of HCM carry sarcomere gene mutations classified as or presumed to be pathogenic [8,9,10,11]. Consistent with this, a recent study demonstrated that isolated and sporadic HCM cases in which the proband does not carry any known HCM mutations or a family history of HCM account for up to 40% of the HCM cases [12], together suggesting that sarcomere dysfunction may be a necessary component but not a required initiating event in HCM pathogenesis. The etiology of HCM is thus likely multifactorial rather than strictly genetic [9,13]. This is most evident by the observations that family and patients, albeit with same genetic mutations, often exhibit a wide range of disease manifestation [13,14]. In fact, in a large dataset of 203 adult gene carriers, none developed cardiac symptoms in the follow-up period, with only 10% converting to HCM clinically [15]. The variable penetrance implies that most HCM gene carriers experience low risk of developing pathological phenotypes over their lifetime. These data underscore the principle that HCM pathogenesis perhaps should not be viewed entirely through the lens of the single-gene-single-disease paradigm but rather better understood as a result of complex interactions between genetic (sarcomere and non-sarcomere gene mutations) and non-genetic (environmental signals and epigenetics) factors.

**Figure 1:**
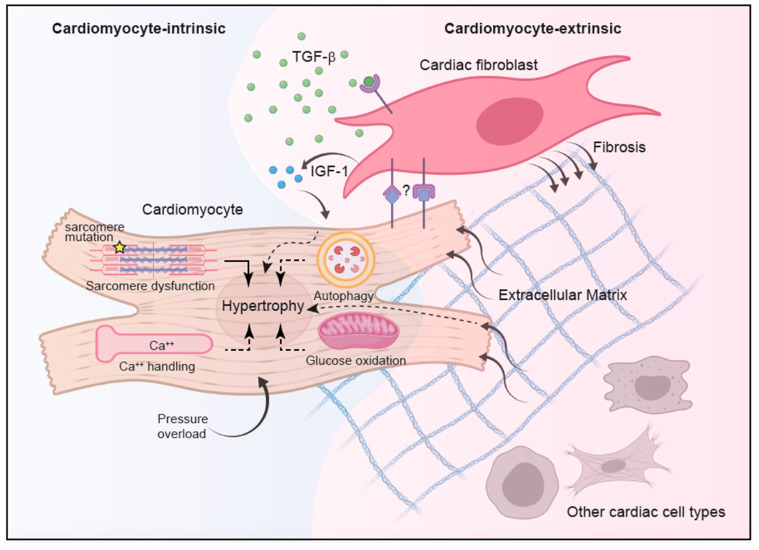
Integrated model of HCM pathogenesis. HCM pathogenesis involves complex interplay between cell-intrinsic processes and intercellular communications. While genetic mutations in sarcomere proteins represent the most well-established cause of cardiomyocyte hypertrophy, alterations in other cellular processes, such as enhanced calcium signaling, reduced autophagy, and suppressed glucose oxidation in response to pressure overload, have also been implicated. With the advance of single-cell transcriptome analysis, better characterization of other non-cardiomyocyte cell types revealed complex intercellular communications during homeostasis and disease states, implying a contribution from non-cardiomyocytes in propagating HCM. In particular, cardiac fibroblasts have been implicated in promoting cardiac fibrosis during HCM in a TGF-β-dependent manner although the source of TGF-β is still unknown. The TGF-β-activated cardiac fibroblasts in turn acquire hypertrophy-inducing capacity and in conjunction with altered extracellular matrix can modify cardiomyocyte growth and function in part through the secretion of IGF-1. Recent data also implicated a role of direct interaction between cardiac fibroblasts and cardiomyocytes in HCM pathogenesis, with the contribution from other cardiac cells remaining elusive.

Mechanistically, at the molecular level, comprehensive characterization of animal models carrying various sarcomere gene mutations demonstrated dysfunctional cardiomyocyte excitation-contraction coupling as a driver of cardiomyocyte hypertrophy [16]. However, whether other HCM-associated non-sarcomere gene mutations also promote cardiomyocyte hypertrophy through alteration in excitation-contraction coupling is not fully understood. Furthermore, at the cellular level, how discrete gene mutations invariably lead to cardiac fibrosis, cardiomyocyte disarray, and asymmetric hypertrophy has not been explained. It is perhaps conceivable that an inciting cellular event, regardless of its genetic or environmental nature, disrupts certain intracellular biological processes with ensuing cardiomyocyte hypertrophy; this in turn alters the extracellular milieu, which subsequently promotes tissue reorganization in an area of otherwise intact cardiomyocytes, culminating in diffuse cardiomyocyte disarray, cardiac fibrosis, and asymmetric septal hypertrophy (Figure 1). In this review, we summarize recent advances on HCM pathogenesis with a particular focus on sarcomere-independent mechanisms.

## 2. Genetic and Non-Genetic Risk Modifiers

Although HCM has been classically considered a Mendelian disease, genetic studies have only identified causal rare variants in less than half of the cases [17] with variable penetrance and expressivity in families and cohorts carrying such mutations [18], suggesting the presence of unidentified genetic and non-genetic risk factors in governing HCM pathogenesis. A recent well-powered metanalysis of three case control genome-wide association studies (GWAS) identified 14 novel loci-associated HCM and demonstrated that a significant portion of HCM risk can in fact be attributed to these common variants [19]. Notably, a polygenic risk score derived from non-sarcomere common variants stratifies disease-free survival in carriers of sarcomere mutations. In support of this, another GWAS study by Harper et al. identified similar common variants in sarcomere mutation negative HCM patients from which a risk score was derived and used to predict phenotypic severity in sarcomere variant carriers [20]. These two seminal studies thus provide the first evidence that a polygenic risk score based on HCM susceptibility variants may explain the interpersonal variability in disease severity among carriers of rare disease-causing variants.

Intriguingly, several HCM-associated variants were also found in patients with dilated cardiomyopathy (DCM) although mostly with opposing effects [19]. Two of the new HCM loci, HSPB7 and BAG3, which have been previously associated with DCM, were moderately protective for HCM in the study. Another locus near TTN, in contrast, increases the risk of both HCM and DCM. Thus, it appears that same genetic pathways may lead to distinct diseases with opposing genetic effects, implying that the disease-modulating effects of the reported common variants may depend on other pre-existing host factors. Tadros et al. described the left ventricular (LV) trait as one of such host factors with HCM-enforcing effects positively associated with LV wall thickness and LV ejection fraction while DCM-enforcing effects correlated with increased LV end systolic and end diastolic volumes. Furthermore, elevated diastolic pressure represents yet another substantial risk for developing HCM [20]. Collectively, these studies convincingly demonstrated the polygenic nature of HCM pathogenesis and revealed the complex interaction between common variants and modifiable host factors in modulating HCM disease progression.

Currently, genetic testing for HCM primarily focuses on detecting mutations in sarcomere genes, as carriers of pathogenic sarcomere variants exhibit a two-fold increased risk for adverse outcomes compared to those without such mutations [21]. For HCM patients with a negative sarcomere gene panel, despite being the majority and with higher incidence of ventricular outflow tract obstruction as well as apical HCM [22], genetic cascade testing is not possible. Rather, US guidelines recommend clinical screening with long-term follow-up of unaffected first-degree relatives [23]. Recommendations for clinical management also do not distinguish between sarcomere-positive and -negative HCM. With recent identification of common variants and non-genetic factors for HCM, expanded genetic screening panel, subsequent risk stratification and risk-based managements may substantially improve the clinical outcomes of both sarcomere-positive and -negative HCM patients.

## 3. Cell-Intrinsic Mechanisms of Cardiomyocyte Hypertrophy

Cardiomyocyte hypertrophy is a defining feature of HCM, with its presence detected early during HCM pathogenesis. Cellular hypertrophy is generally deemed as a cell’s natural response when the work produced does meet the organ’s demand. In cardiomyocytes, it has been repeatedly demonstrated that suboptimal contractile machinery, such as in the case of sarcomere dysfunction, is consistently associated with hypertrophy of the affected cells. Similarly, metabolic alterations resulting in insufficient supply of biological fuel has also been shown to induce cellar hypertrophy. Conversely, blockade of growth factor signaling through a pharmacologic approach or nutrient starvation appears to ameliorate the hypertrophic phenotype. Because the molecular mechanisms underlying sarcomere gene mutation-mediated HCM have been reviewed extensively elsewhere [24], in this section, we highlight several novel mechanisms of cardiomyocyte hypertrophy that do not necessarily require alteration in excitation-contraction coupling.

### 3.1. Calcium Signaling

Dysregulated calcium handling is often co-observed in cardiomyocytes harboring sarcomere gene mutations [25], with prolonged activation of calcium-calmodulin dependent protein kinase II (CaMKII) implicated to play a major role [26,27]. However, perturbation in calcium signaling has also been observed in HCM patients without sarcomere gene mutations [27]. Notably, unlike CaMKII activation, mRNA expression of sarcoendoplasmic reticulum calcium transport ATPase (SERCA2A) as well as the SERCA2A/phospholamban ratio were reduced regardless of sarcomere genotypes. Additionally, enhancing calcium signaling in cardiomyocytes via calcineurin or NFAT3 overexpression alone is sufficient to drive cardiomyocyte hypertrophy both in vitro and in vivo [28,29], together suggesting that sustained calcium signaling may be an independent trigger for hypertrophy, one that precedes sarcomere dysfunction. While the exact nature of the signals that trigger increased calcium signaling is unclear, a seminal study by Kumarapeli et al. demonstrated a critical role of the chaperone protein αB-Crystallin (CRYAB) in suppressing nuclear factors of activated T-cell (NFAT)-driven cardiomyocyte hypertrophy both in vitro and in vivo [30]. Mechanistically, CRYAB appears to prevent nuclear localization of NFAT. Notably, two pathogenic variants of CRYAB, R120G and R123W, were identified in patients with proteotoxic cardiomyopathy [31] and in a pair of adult monozygotic twins with HCM [32], respectively. Whereas subsequent animal studies demonstrated that overexpression of the CRYAB^R120G^ pathogenic variant induces sarcomere dysfunction by triggering desmin aggregation, this phenotype was not seen in CRYAB^R123W^-expressing cardiomyocytes (unpublished data). Whether the CRYAB^R123W^ variant instead drives cardiomyocyte hypertrophy by enhancing calcium signaling remains to be tested. Nevertheless, altered calcium handling represents a sarcomere-independent mechanism of cardiomyocyte hypertrophy, with CRYAB being a potential key nodal regulator.

### 3.2. RAS Pathway

In addition to altered calcium signaling, congenital mutations in genes encoding components of the RAS/mitogen-activated protein kinase (MAPK) pathway are also linked to HCM as a part of the developmental disorders, collectively called RASopathies [33,34]. Patients with RASopathies, such as Noonan, Costello, and cardio-facio-cutaneous syndromes, are typically born with facial dysmorphism, skeletal defects, and later in life develop various types of cardiomyopathies among which HCM is the most common myocardial pathology, represents poor prognosis and is the leading cause of death [35,36,37]. Mechanistically, activation of the RAS/RAF/MEK/MAPK pathway appears to de-repress the fetal cardiac gene expression program in mice, resulting in upregulation of fetal genes, such as beta-myosin heavy chain and atrial natriuretic peptide, as well as downregulation of SERCA, culminating in cellular hypertrophy [38].

Many clinically relevant mutations have been identified in RASopathies with missense mutation in protein tyrosine phosphatase, with non-receptor type 11 (PTPN11) reported in nearly 50% of Noonan syndrome patients [39] and up to 65% of patients who later develop HCM harboring gain-of-function mutations in RAF1 [39,40,41]. Indeed, mice heterozygous for the gain-of-function *Raf1^L613V^* allele faithfully recapitulated Noonan syndrome, including HCM with extensive cardiomyocyte hypertrophy and cardiac fibrosis [42]. Molecular characterization of the affected cardiac tissues revealed a change in myosin heavy-chain isoform with an increased β-MHC to α-MHC ratio, consistent with reactivation of the fetal sarcomere gene program, a hallmark of pathological hypertrophy. Not surprisingly, profiling of the RAS/MAPK pathway showed enhanced activation of MEK and ERK, with activation of other MAPK, JNK, and p38 remaining unaltered. Notably, post-natal treatment of *Raf1^L613V^* allele-carrying mice with an MEK inhibitor was sufficient to block HCM development, thus demonstrating a causal relationship between the hyperactivated RAS/MAPK pathway and HCM pathogenesis.

As one of the most commonly mutated genes in RASopathies, PTPN11 encodes SHP2, a non-receptor tyrosine phosphatase, which plays positive regulatory roles in signal transduction, particularly in the RAS pathway. Cell culture experiments suggest a gain-of-function effect of Noonan syndrome-associated PTPN11 variants, primarily through releasing auto-inhibition of SHP2 domain activity, resulting in heightened ERK1/2 signaling. While mice heterozygous for the *Ptpn11^Y279C^* allele readily recapitulated several hallmark features of Noonan syndrome, including cardiomyocyte hypertrophy, disarray, and fibrosis, in contrast to mice carrying the *Raf1^L613V^* allele, ERK1/2 activation was in fact attenuated in *Ptpn11^Y279C/+^* heart tissues both at the basal state and in response to agonist stimulation [43]. Intriguingly, the mTOR/AKT pathway was hyperactivated with post-natal blockade of mTOR signaling by rapamycin sufficient to normalize HCM in *Ptpn11^Y279C^* heterozygous mice.

The discrepancy between these two studies raises an important issue regarding mechanistic studies of HCM in RASopathies. While mice carrying germ-line mutations in RASopathy-associated genes indeed recapitulate HCM phenotypes, whether these variants exert their effects in a cardiomyocyte-intrinsic manner cannot be formally established. Although in vitro experiments consistently demonstrated that a hyperactive RAS-MAPK-MEK-ERK pathway can drive cardiomyocyte hypertrophy, the effects of ERK activation in other cell types and their impacts on cardiomyocytes in vivo should not be overlooked. This is most evident by a study demonstrating that cardiomyocyte-specific expression of a gain-of-function RAS variant, *Kras^G12D^*, in fact did not result in HCM despite the neonatal cardiomegaly phenotype seen in mice with germ-line expression of *Kras^G12D^*, suggesting a role of non-cardiomyocytes in driving RASopathies-associated HCM [44]. Thus, to what extent gain-of-function *Raf1* variants drive HCM in a cardiomyocyte-intrinsic manner should be re-assessed using mice with cardiomyocyte-restricted expression of Raf1 mutants. Likewise, whether gain-of-function *Ptpn11* variants directly activate the mTOR/AKT pathway in cardiomyocytes should also be scrutinized with more precise genetic models. A recent proteomic study of plasma from HCM patients and control individuals indicated systemic activation of the RAS-MAPK pathway in HCM, consistent with global rather than cell-specific activation [45].

### 3.3. Autophagy

Autophagy is an evolutionarily conserved cellular process in which intracellular macromolecules and organelles undergo regulated degradation to generate reusable, biologically active monomers. As such, autophagy is often activated in response to cellular stress: both extracellular signals, such as nutrient deprivation, and intracellular stimuli, such as accumulation of misfolded proteins, have been shown to trigger autophagy. Notably, autophagy is suppressed in cardiomyocytes isolated from HCM patients with MYBPC3 mutations and this defect was reproduced in mice carrying knock-in mutations of the Mybpc3 gene [46]. Importantly, administration of rapamycin, which principally activates autophagy, is sufficient to ameliorate cardiomyocyte hypertrophy, suggesting that during cardiomyocyte stress, autophagy, albeit reduced, is necessary to prevent pathologic hypertrophy. Intriguingly, early studies using inducible models of pathological hypertrophy demonstrated that increasing pressure overload can lead to a reduction in autophagy flux and subsequent cardiomyocyte hypertrophy [47,48]. Furthermore, such pathologic hypertrophy is markedly worsened when autophagy is abolished via cardiomyocyte-specific deletion of ATG5, a protein required for autophagy [49]. Similar results are seen in several independent genetic models in which other key regulators of the autophagy pathway are disrupted [50,51,52]. Collectively, these findings strongly suggest that a dysregulated autophagy response contributes to HCM pathogenesis in a sarcomere-independent manner. Notably, at steady state, loss of ATG5 per se does not result in detectable pathological changes in cardiomyocytes, exemplifying the notion that HCM pathogenesis may be driven by the concerted action of genetic predisposition and environmental triggers with diminished autophagy response as a potential susceptibility factor rather than an initiator.

### 3.4. Glucose Metabolism

Biofuel utilization by the heart varies by age and circumstance, with glucose as a preferred energy source during neonatal periods and fatty acids being favored by post-natal hearts [53]. Strikingly, when under stress, such as an increased pressure load, rising intracellular AMP activates the AMP-activated protein kinase, which subsequently promotes a switch of metabolism from fatty acid oxidation back to glycolysis [54,55]. In fact, increased glucose uptake by the heart can be observed as early as one day after transverse aortic constriction (TAC) before detectable cellular hypertrophy [56], suggesting that the switch to glycolytic metabolism represents an adaptive response to pressure overload rather than a consequence of cellular hypertrophy. Interestingly, glucose utilization in response to pressure overload appears to exert a cardio-protective effect, delaying pathologic hypertrophy, as mice with suppressed glycolysis in cardiomyocytes exhibit a markedly exaggerated hypertrophic response compared to wild-type controls following TAC [57,58]. However, despite its initial cardio-protective effects, sustained glycolysis is often co-observed in hypertrophic hearts. This paradoxical effect is likely due to impaired oxidation of glucose, resulting in uncoupling of glucose uptake from its utilization in the mitochondria [55]. Consistent with this notion, a recent study showed that the expression levels of mitochondrial pyruvate carriers (MPCs), which import pyruvate into mitochondria for oxidation, are suppressed in hearts from heart failure patients as well as murine hearts following TAC [59]. Importantly, mice with cardiomyocyte-specific MPC deficiency or knock-down develop accelerated cardiac hypertrophy compared to wild-type controls. Conversely, overexpression of MPCs in cardiomyocytes markedly ameliorates cardiac hypertrophy and dysfunction. Glucose oxidation and metabolism are known to be impaired in HCM patients [60], although myocardial uptake is increased and fatty acid utilization is reduced [61]. Together, these findings suggest that as an adaptive mechanism, stress-induced upregulation of glycolytic metabolism provides an initial cardio-protective effect, which is subsequently rendered pathogenic by the unmatched capacity of mitochondria to oxidize glucose. While the precise mechanisms underlying mitochondrial dysfunction are unclear, these studies nevertheless establish altered glucose metabolism as another potential regulatory node in HCM pathogenesis.

### 3.5. Non-Sarcomere Structural Proteins

While sarcomere gene mutations account for the majority of HCM-associated rare variants, mutations in other non-sarcomere structural proteins have also been reported in HCM patients. Titin (TTN), the largest protein of the human genome, is one of the essential components of the sarcomere complex. Structurally, TTN serves as a molecular spring spanning half-way through the sarcomere, tethered to the Z-disk on one end and M-line on the other. In addition to providing structural support, TTN has also been implicated as a mechanosensor and in signal transduction [62]. While classically associated with DCM [63], missense and frameshift mutations in TTN have also been reported in sporadic cases of HCM [64,65,66]. In a small cohort of the Chinese population, while the frequency of TTN mutation is comparable between HCM patients and healthy controls, TTN mutation carriers are more likely to experience cardiovascular death [65], suggesting a disease-modifying rather than causal role of TTN variants. Notably, approximately half of the HCM patients in the study also carry mutations in sarcomere genes, further implying a potential synergy between TTN and sarcomere variants in HCM pathogenesis.

The mechanisms by which TTN variants contribute to cardiac hypertrophy is controversial. A recent study reported hyperphosphorylation of TTN in HCM patients and implicated phosphorylation of TTN by protein kinase D (PKD) as a potential regulatory mechanism of cardiomyocyte contractility [67]. While PKD ablation in cardiomyocytes appears to be protective against pressure overload-induced cardiac hypertrophy and fibrosis [68], to what extent such an effect is due to TTN hypophosphorylation remains unclear as the contribution from other PDK substrates cannot be completely ruled out. Nevertheless, recent large-scale GWAS studies also identified TTN as one of the common variants associated with HCM in addition to DCM [19,20]. Whether TTN variants represent an independent risk factor remains to be investigated.

Lamin A/C (LMNA), as an integral component of the nuclear membrane, maintains nuclear stability and more recently has also been implicated to ensure structural integrity of the whole cell through interactions with nuclear lamina, cytoskeleton, and the extracellular matrix (ECM) [69,70]. The association between LMNA mutations and DCM has been long established, with LMNA variants accounting for up to a third of heritable DCM cases [71]. However, not unlike BAG3 and HSP7 variants, which exert opposing effects on HCM and DCM progression, mice heterozygous for a null mutation in the *Lmna* allele do develop DCM over time but more strikingly are partially protected from left ventricular hypertrophy in a pressure-overload model [72]. Mechanistically, partial loss of *Lmna* blunts the hypertrophic response in part by inhibiting transcription of the mechanosensory gene, *Erg1* [72], while complete loss of *Lmna* leads to cardiomyocyte shortening and decreased contractility, resulting in rapid development of DCM. Interestingly, whereas most missense mutations in the LMNA gene are exclusively associated with DCM, two novel LMNA variants, C591F and R644C exhibit pleiotropic effects, with mutation carriers developing a wide spectrum of cardiomyopathies, including both DCM and HCM [73,74], implying external stimuli as a potential pathogenic co-driver. These findings thus suggest that *Lmna* may in fact function as a rheostat through which mechanotransduction pathways and pathophysiological adaptions integrate to impact the direction of disease progression.

## 4. Cell-Extrinsic Mechanisms of HCM

Extensive cardiac fibrosis and cardiomyocyte disarray are the hallmarks of HCM, distinguishing it from other etiologies of left ventricular hypertrophy, for instance, one caused purely by pressure overload. Diffuse peri-cardiomyocyte fibrosis and later stage fibrotic replacement of cardiomyocytes are hypothesized to promote clinical complications of HCM. However, the exact causes of fibrosis remain controversial and whether such fibrotic response, if at all, impacts cardiomyocyte function is also poorly understood. In this section, we summarize the current understanding of how changes in the cardiac microenvironment may impact cardiomyocyte hypertrophy.

The cellular composition of the heart at steady state is heterogenous, comprising of at least five distinct cell types, with cardiac fibroblasts representing ~20% and ~30% of total cells in mice and humans, respectively [75,76]. The cardiac fibroblasts have been implicated as mediators of cardiac fibrosis in response to ischemic injury in a transforming growth factor (TGF)-β-dependent manner. Notably, cardiac fibroblast activation was also observed in a murine model of pressure overload hypertrophy [77] and hearts from HCM patients were enriched for TGF-β transcripts compared to healthy controls [78], together implying a potential role of cardiac fibroblasts and TGF-β in cardiac fibrosis during HCM pathogenesis. Indeed, in animal models, administration of TGF-β receptor blocking antibody is sufficient to delay the development of fibrosis [79], with inducible ablation of TGF-β receptor signaling specifically in cardiac fibroblasts resulting in a similarly reduced fibrotic response following TAC [80]. Collectively, these findings identified the cardiac fibroblasts-TGF-β axis as a potential mechanism underlying cardiac fibrosis during HCM pathogenesis. Evidence for global TGF-β activation is also found in plasma from HCM patients compared to controls [45]. Another recent study has implicated a JAK2-STAT3-COL4A2 pathway as a driver of the fibrotic phenotype that could distinguish individual HCM patients on the basis of fibrosis severity [81], and additional exploration of cardiac fibroblast function and activation in HCM in human tissue and experimental models are likely to yield additional candidate pathways.

Despite its strong association with HCM, how cardiac fibrosis contributes to HCM pathogenesis is poorly understood. While in vivo genetic studies are lacking, results using in vitro co-culture systems have provided some insights. Intriguingly, normal cardiomyocytes undergo hypertrophy when co-cultured with fibroblasts isolated from post-TAC hearts and this response is abolished by a small molecule inhibitor against TGF-β signaling [82], raising the possibility that an altered peri-cardiomyocyte microenvironment may in fact be the driver of hypertrophic responses in an area of otherwise healthy cardiomyocytes. Conceivably, the spatial heterogeneity in fibroblast responses would then account for the often-observed asymmetric nature of cardiac hypertrophy.

Although cardiac fibroblasts appear to require intact TGF-β signaling to drive cardiomyocyte hypertrophy, whether direct interaction with cardiomyocytes is necessary for this effect is controversial. Whereas insulin-like growth factor 1, a potent inducer of cellular growth, has been implicated as one of the soluble mediators produced by activated cardiac fibroblasts [83], another study indicated a role of insoluble extracellular matrix from HCM hearts in promoting abnormal contractile function in genetically normal cardiomyocytes [84]. Notably, fibronectin, a large ECM-associated glycoprotein, appears to promote HCM pathogenesis. Inducible attenuation of fibronectin expression partially protected mice from ventricular hypertrophy after TAC [85]. Mechanistically, engagement of fibronectin triggers nuclear localization of NFAT, which in turn promotes re-acquisition of the fetal cardiac gene expression program. Because the genetic model non-selectively ablates fibronectin in most somatic cells, the exact source of fibronectin remains elusive, although a more recent study, by inducibly abolishing fibronectin specifically in fibroblasts, suggests fibroblast-derived fibronectin as a major driver for cardiac hypertrophy and fibrosis after ischemic injury [86]. In patients with HCM, the plasma concentration of fibronectin is significantly, albeit mildly, reduced compared to healthy controls [87]. Given that the plasma fibronectin may constitute up to 60% of the total fibronectin content [88], significant ECM formation and fibrosis may contribute to consumption of fibronectin from the circulating pool. Alternatively, altered activity of metalloproteinase and their inhibitors have been associated with HCM [89,90], which may also impact circulating fibronectin levels. Collectively, these data suggest a reinforcing role for the extracellular matrix in promoting myocyte contractile dysfunction and HCM pathogenesis. Thus, it is conceivable that interactions between fibroblasts and cardiomyocytes either directly or through the extracellular matrix contribute to the hypertrophic response of the latter and to the fibrotic response of the former.

In addition to cardiac fibroblasts, pro-inflammatory macrophages in the cardiac tissues have also been implicated to promote HCM. Single-cell transcriptomic analysis of murine heart tissues upon pressure overload revealed upregulation of activation markers and several pro-inflammatory cytokines in cardiac tissue macrophages [77]. Notably, similar patterns of alterations were also observed in human hearts with HCM compared to normal counterparts. Intriguingly, pharmacologic inhibition of inflammation by dapagliflozin, albeit non-specific, reduced macrophage activation and cardiac fibrosis. Mechanistically, conditioned medium from cardiac tissue macrophages post pressure-overload was sufficient to induce expression of hypertrophy-related genes in otherwise normal cardiomyocytes. These data collectively imply inflammatory mediators as another potential perpetrator of HCM pathogenesis. To what extent this inflammatory response is triggered by cardiomyocyte dysfunction and damage remains unclear. Future studies to define the reciprocal communication pathways between cardiomyocytes and non-cardiomyocytes to infer potential mechanisms of HCM pathogenesis will likely provide novel insights.

Aside from cardiac fibroblasts and inflammatory macrophages, the roles of other cardiac cell types in HCM pathogenesis are unclear. Thanks to the advance of single cell transcriptomic analysis, the full repertoire of human cardiac cells has only recently been realized, with the lineage identity of each respective constituent becoming more refined [76,91,92]. While exactly how each population and their interactions contribute to HCM pathogenesis awaits further investigation, single-cell receptor-ligand pair gene expression analysis using murine hearts has been fruitful and provided us with inferred cellular interactomes [77,93]. Applying network analyses to hearts from HCM patients may thus help uncover pathogenic intercellular interactions and disease-driving signaling pathways. These studies will not only expand our understanding of HCM pathogenesis beyond sarcomere-centric views but also may reveal novel and accessible extracellular targets for therapeutic intervention.

## 5. Conclusions

Emerging evidence suggests that the pathogenesis of HCM may be more complicated than postulated under the conventional single gene-single disease paradigm. Rather, dysregulated interactions between cell-intrinsic and -extrinsic pathways as well as altered cell–cell interaction may in fact underly HCM pathogenesis at the tissue level. Notably, under this model, the initial inciting molecular event may occur in any cells in addition to cardiomyocytes, although the experimental models thus far invariably assumed a cardiomyocytic origin. To date, the precise mechanisms by which initial alteration in intracellular biological processes modifies the interactions of the affected cell with its neighbors are still incompletely understood. Although in vitro culture experiments using explanted cardiac tissues from animals with HCM demonstrated that HCM-conditioned extracellular milieu is sufficient to modify contractile functions of an otherwise healthy cardiomyocyte [84], to what extent such a phenomenon occurs naturally in vivo remains untested. Recently, we performed single-nucleus RNA sequencing analyses on explanted hearts from HCM patients without known sarcomere mutations and indeed observed altered cellular interactomes with significantly dysregulated expression of genes involved in extracellular matrix synthesis in cardiac fibroblasts as well as pathways associated with direct cell–cell interaction (unpublished data). While single-cell transcriptome profiling offered valuable insights into the potential biological function of the dysregulated pathways, animal models that allow for spatially and temporally regulated induction of HCM-driving mutations followed by longitudinal profiling of transcriptomes and cellular interactomes may be necessary to unveil the inciting molecular events that set forth the disease-propagating cascade.

## Data Availability

Not applicable.

## Abbreviations

HCM	Hypertrophic Cardiomyopathy
GWAS	Genome Wide Association Study
DCM	Dilated Cardiomyopathy
LV	Left Ventricle
CaMKII	Calcium-calmodulin Dependent Protein Kinase II
SERCA2A	Sarcoendoplasmic Reticulum Calcium Transport ATPase
CRYAB	αB-Crystallin
NFAT	Nuclear Factor of Activated T-cells
TAC	Transverse Aortic Constriction
MPC	Mitochondrial Pyruvate Carrier
TTN	Titin
PKD	Protein Kinase D
LMNA	Lamin A/C
ECM	Extracellular Matrix
TGF-β	Transforming Growth Factor-β
